# Effects of different tooth movement patterns and aligner thicknesses on maxillary arch expansion with clear aligners: a three-dimensional finite element study

**DOI:** 10.3389/fbioe.2024.1424319

**Published:** 2024-06-25

**Authors:** Na Li, ChunJuan Wang, Min Yang, DingGen Chen, MingYuan Tang, DaoKun Li, ShengLei Qiu, Qi Chen, Yi Feng

**Affiliations:** ^1^ Silk Crossing Clinic, Affiliated Hospital of North Sichuan Medical College, Nanchong, China; ^2^ Department of Stomatology, North Sichuan Medical College, Nanchong, China; ^3^ Chongqing Key Laboratory of Oral Diseases, Chongqing Municipal Key Laboratory of Stomatological Biomedical Engineering, Chongqing, China

**Keywords:** clear aligner, arch expansion, finite element analysis, tooth movement pattern, thickness

## Abstract

**Objectives:**

The objective of this study was to investigate the biomechanical effects of different tooth movement patterns and aligner thicknesses on teeth and periodontal tissues during maxillary arch expansion with clear aligners, to facilitate more precise and efficient clinical orthodontic treatments.

**Methods:**

Three-dimensional models including teeth, maxilla, periodontal ligament, and aligner were constructed and subjected to finite element analysis. Tooth displacement trends and periodontal ligament stresses were measured for seven tooth displacement patterns (divided into three categories including overall movement of premolars and molars with gradually increasing molar expansion in each step; distributed movement of premolars and molars; and alternating movement between premolars and molars at intervals) and two aligner thicknesses (0.5 mm and 0.75 mm) during maxillary arch expansion with clear aligners.

**Results:**

When expanding the maxillary arch with clear aligners, the effective expansion of the target teeth mainly showed a tilting movement trend. Increasing the amount of molar expansion increased the buccal displacement of the first molar but decreased the buccal displacement of the premolars. The mean buccal displacement of the target teeth was greater in the posterior teeth interval alternating movement group (0.026 mm) than in the premolar/molar distributed movement group (0.016 mm) and the overall movement group (0.015 mm). Increasing aligner thickness resulted in greater buccal displacement of the crowns and increased stress on the periodontal ligaments.

**Conclusion:**

Increasing the amount of molar expansion reduces the efficiency of premolar expansion. Alternating movement of premolars and molars at intervals achieves a higher arch expansion efficiency, but attention should be paid to the anchorage of adjacent teeth. Increasing the thickness of the aligner increases the expansion efficiency but may also increase the burden on the periodontal tissues.

## 1 Introduction

In recent years, with the rapid development of digital technology and materials, clear aligners have been favored by orthodontists and patients due to their advantages such as aesthetics, comfort, convenience, hygiene, and prospective treatment design, and have been widely used to correct various malocclusions (Gao et al., 2021; Shokeen et al., 2022). Clear aligners are typically made of thermoformed plastic polymers that, when worn in the mouth, deform to generate resilient forces that are transmitted to the teeth and periodontal tissues to promote tooth movement and tissue remodeling (Ma and Li, 2021; Bichu et al., 2023). Through small-scale sequential movements, it eventually moves the teeth to the desired positions (Cortona et al., 2020). However, its efficiency and precise control over tooth movement remain suboptimal, particularly regarding bodily movement where unpredictability is a common issue (Simon et al., 2014).

Maxillary transverse deficiency is a common malocclusion in orthodontic clinical practice, with an incidence of 8%–23% in the deciduous and mixed dentition and approximately 10% in the permanent dentition (MacGinnis et al., 2014; Brunetto et al., 2017). It can manifest as varying degrees of narrow dental arches, mismatched upper and lower dental arch relationships, posterior crossbite, crowding of the dental arch with protrusion, excessive buccal corridors when smiling, leading to malocclusion and even restricting the growth and development of the jaw bones, affecting the function and aesthetics of the stomatognathic system (Pithon et al., 2014). In such cases, arch expansion is often required. Recently, clear aligners have been proven to be effective tools for achieving maxillary dental expansion to improve smile aesthetics, obtaining space to resolve mild to moderate dental crowding, and correcting dental crossbites (D’Antò et al., 2023). However, clear aligners still have certain limitations in terms of dental arch expansion: on the one hand, the expansion efficiency gradually decreases from the anterior to the posterior dental arch, and the molar expansion efficiency is still not ideal (Lione et al., 2021); on the other hand, an obvious buccal inclination of the teeth often occurs during expansion with clear aligners (Zhou and Guo, 2020). Buccal inclination of the posterior teeth can cause the palatal cusp to droop, resulting in loose occlusion and occlusal interference during mandibular functional movements, thus causing chewing discomfort and temporomandibular joint discomfort in patients. At the same time, drooping of the palatal cusp leads to an increase in the occlusal plane angle and mandibular clockwise rotation, worsening the adverse facial profile in patients with vertical growth pattern. Excessive buccal inclination of the teeth may also lead to buccal bone fenestration and gingival recession, damaging the health of periodontal tissues. In order to improve the efficiency of tooth movement with clear aligners and achieve precise control to establish a functional stomatognathic system, research on the biomechanical mechanism of clear aligner systems is particularly important. At present, only a few studies have investigated the biomechanical effects of clear aligners during expansion, and the biomechanical mechanisms under different designs are still unclear.

The finite element method (FEM) is a mathematical simulation method used to calculate the stress and deformation of complex structures under external forces. It allows for comprehensive and realistic three-dimensional biomechanical analysis of intraoral tissues and has been widely applied in orthodontic research (Elshazly et al., 2022). FEM is a non-invasive and precise method that provides quantitative and detailed data on the physiological responses occurring within tissues. Through this method, tooth displacement and periodontal stress distribution resulting from orthodontic forces during the correction process can be observed, visualizing tissue responses and guiding more rational clinical treatment designs (Ahmed et al., 2023).

Therefore, this study aims to comprehensively demonstrate, using the finite element method, the biomechanical effects of different tooth displacement patterns and different aligner thicknesses during maxillary arch expansion with clear aligners, within the entire orthodontic system.

## 2 Materials and methods

### 2.1 Construction of the 3D finite element model

This study was approved by the Ethics Committee of the Affiliated Hospital of North Sichuan Medical College (No.2023ER159-1).

**FIGURE 1:**
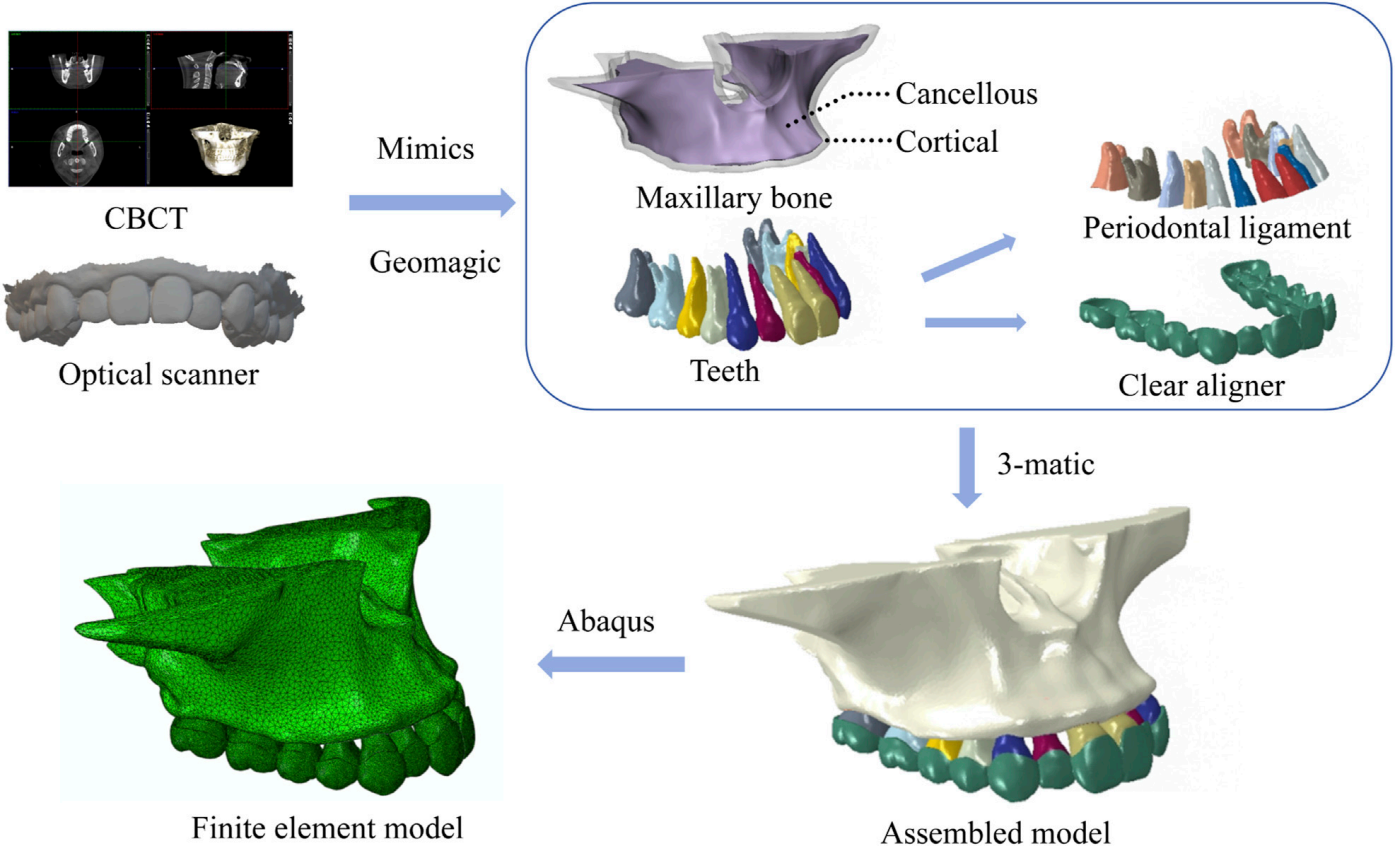
3D models of maxillary bone, teeth, periodontal ligament, assembled model and finite element model.

An adult patient with maxillary dental arch constriction was selected to obtain cone beam computed tomography (CBCT, Planmeca ProMax3D, Finland) and intraoral optical scanning (3 shape, Denmark) data. The CBCT images were stored in the standard digital imaging and communications in medicine (DICOM) format and imported into Mimics19.0 software (Materialise, Belgium) to construct preliminary three-dimensional (3D) models of the maxilla and dentition through threshold separation based on differences in tissue gray values. The 3D models were exported in STL format and imported into Geomagic Studio 2015 software (Geomagic, United States), where the “relaxation” command was used for surface smoothing. Crown data obtained from intraoral scanning were used to fit with the dental model constructed by CBCT to further improve the precision of the dentition modeling (Barone et al., 2017). Cortical and cancellous bone models were created by moving the maxilla inward by 2.0 mm, while the tooth roots were evenly expanded outward by 0.25 mm (Schmidt and Lapatki, 2019) to construct a periodontal ligament (PDL) model. The clear aligner model was constructed based on the crowns: the bilateral posterior teeth were first moved buccally to reach the desired target positions, and then the crowns were evenly expanded outward by 0.75 mm or 0.5 mm to obtain the aligner model. The components of each model were assembled in 3-matic 11.0 (Materialise, Belgium), and a 3D finite element solid model containing the maxilla, teeth, periodontal ligament, and clear aligner was obtained (Figure 1).

### 2.2 Experiment design

**FIGURE 2:**
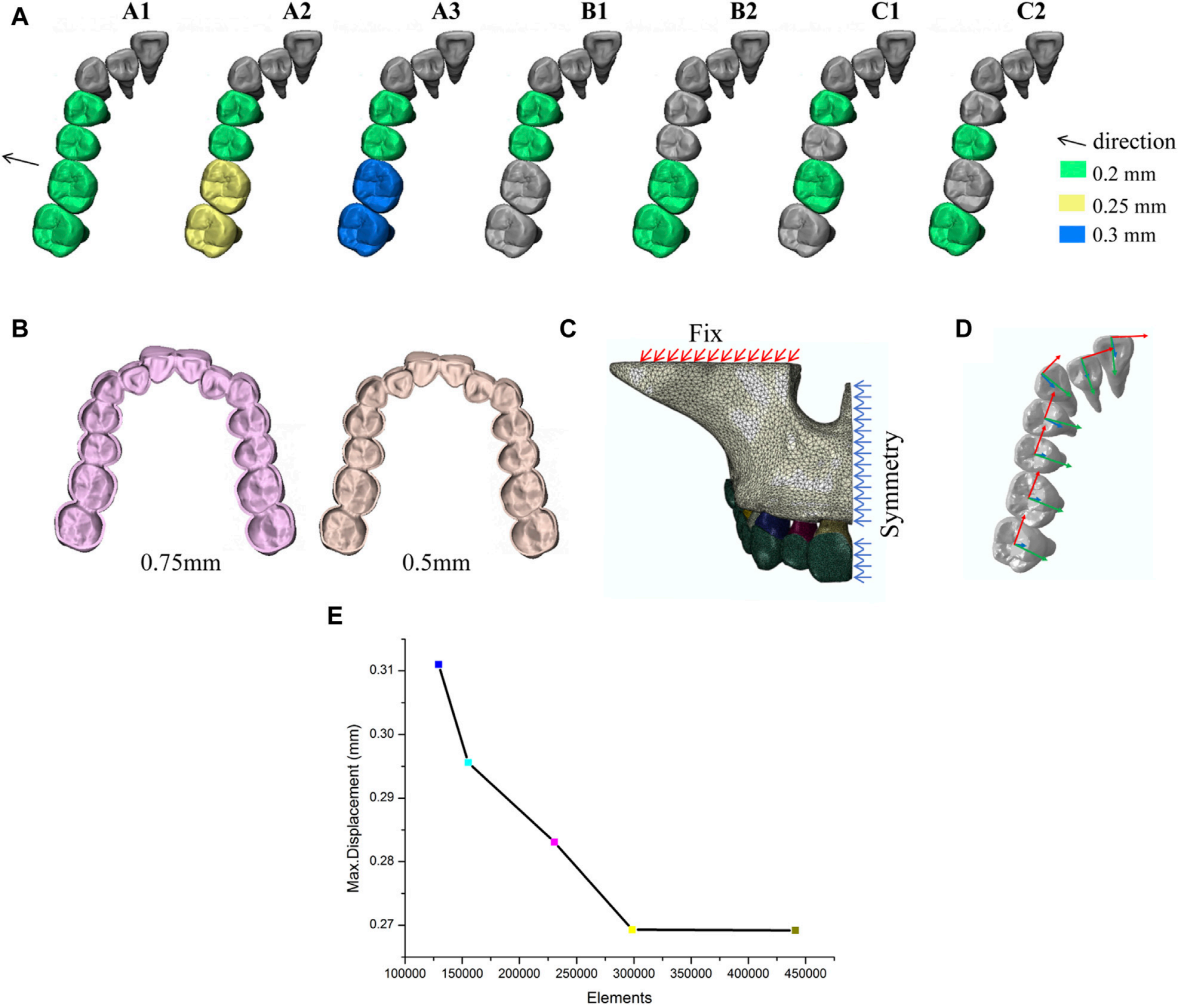
Experiment design. **(A)** Different tooth movement patterns. **(B)** Different aligner thicknesses. **(C)** Boundary constraints. **(D)** Coordinate system setting. **(E)** Mesh convergence validation.

Seven expansion movement patterns were designed for bilateral maxillary posterior dental arch expansion. These patterns were categorized into three types: overall movement where premolars and molars move simultaneously with increasing molar displacement (A1, A2, A3), distributed movement of premolars and molars (B1, B2), and alternating movement between premolars and molars at intervals (C1, C2). Based on the experimental results, a model with the optimal displacement pattern was selected to design two thicknesses of aligner (C1, D). Ultimately, eight different aligner models were obtained. The experimental groups are shown in Table 1 and Figure 2A, B.

**TABLE 1:** Experimental groups.

Group	Target teeth and displacement (mm)	Aligner thickness (mm)
A1	Premolars: 0.2 and Molars: 0.2	0.75
A2	Premolars: 0.2 and Molars: 0.25	0.75
A3	Premolars: 0.2 and Molars: 0.3	0.75
B1	Premolars: 0.2	0.75
B2	Molars: 0.2	0.75
C1	First premolar and first molar: 0.2	0.75
C2	Second premolar and second molar: 0.2	0.75
D	First premolar and first molar: 0.2	0.5

### 2.3 Material properties and meshing

The material properties were set based on previous literature, with the maxilla, teeth, periodontal ligament, and clear aligner all defined as homogeneous, isotropic linear elastic materials (Kim et al., 2020). The material properties (Wang et al., 2012; Gomez et al., 2015) of all components are shown in Table 2. In this study, tetrahedral elements (C3D10M) were used to mesh the models and a mesh sensitivity analysis (Figure 2E) was carried out, which showed that the meshes had converged. The number of elements and nodes for each model are shown in Table 3.

**TABLE 2:** Material properties.

Material	Young’s modulus (MPa)	Poisson’s ratio
Teeth	19,600	0.30
Periodontal ligament	0.69	0.45
Cortical bone	13,700	0.30
Cancellous bone	1,370	0.30
Clear aligner	528	0.36

**TABLE 3:** Number of nodes and elements of the finite element model.

Model	A1	A2	A3	B1	B2	C1	C2	D
Elements	5,76,877	6,02,850	6,04,412	6,04,152	6,02,591	6,03,673	6,03,504	5,86,049
Nodes	10,70,666	11,17,151	11,19,883	11,19,283	11,16,616	11,18,387	11,18,070	10,92,264

### 2.4 Boundary constraints and contact conditions

The orbital base of the maxillary alveolar bone was set as a fixed constraint, and the whole model was set symmetry constraint with the mid-sagittal plane as the symmetry axis (Figure 2C). Bonded contacts were established between teeth and PDL, as well as between PDL and alveolar bone. A nonlinear surface-to-surface contact relationship was defined between the outer surface of the crowns and the inner surface of the clear aligner, with a friction coefficient of µ = 0.2 (Gomez et al., 2015). No additional constraints or loads were applied to the teeth or the clear aligner.

### 2.5 Coordinate system setting

A local coordinate system was established for each tooth, with the center of the occlusal or incisal surface of the crown as the origin, the mesial-distal direction defined as the *X*-axis extending in a positive direction toward the mesial side, the buccolingual direction defined as the *Y*-axis extending in a positive direction toward the lingual side, and the vertical direction defined as the *Z*-axis extending in a positive direction toward the root (Figure 2D).

### 2.6 Calculation and analysis

Nonlinear iterative analyses were conducted using Abaqus 6.14 software (Simulia, France). Due to the basic bilateral symmetry of the model, analysis was focused on the right side. The buccal cusp and lingual cusp of premolars and the mesial-buccal cusp and mesial-lingual cusp of molars, as well as the corresponding root apical points of each of the above points, were selected as landmarks for the crown and root displacement analysis. Analysis index included: 1) initial displacement and displacement trends of teeth; 2) von Mises stress distribution and hydrostatic pressure distribution in PDL.

## 3 Results

### 3.1 Effects of different tooth movement patterns

#### 3.1.1 Initial displacement and displacement trends of teeth

To ensure consistency in treatment time, when comparing the tooth displacement between overall movement and distributed movement patterns, the mean tooth displacement for each model in the distributed movement groups was used for analysis and comparison.

**FIGURE 3:**
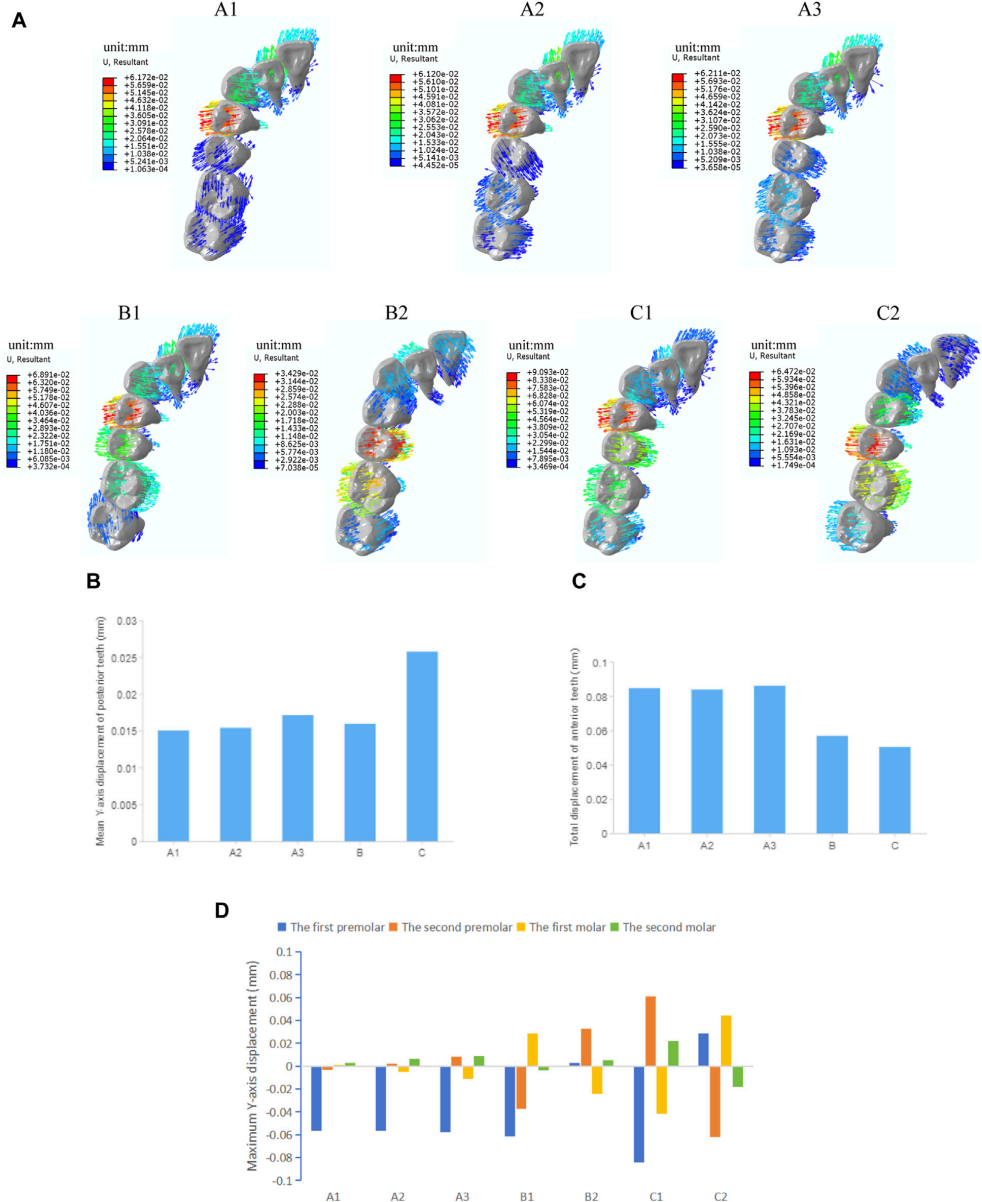
Initial displacement trends and displacement of the maxillary teeth in groups with different tooth movement patterns. **(A)** Initial displacement trends. **(B)** Mean *Y*-axis displacement of posterior teeth. **(C)** Total displacement of anterior teeth. **(D)** Maximum *Y*-axis displacement of posterior teeth.

As shown in Figure 3A, the effective expansion of the target teeth is characterized by a tilt movement trend, with the crowns moving buccally and the roots moving palatally. The displacement of the crowns exceeds that of the roots, accompanied by a tendency for the crowns to move distally. However, not all target teeth show a buccal movement tendency. The posterior teeth, which were not designed to move, serve as anchorage units and show a tilt movement trend with the crowns moving toward the palatal side. In overall movement groups (A), two target teeth did not achieve effective buccal movement and some even showed an opposite palatal movement trend. When the posterior teeth are moved by the same amount overall, the buccal displacement of the teeth gradually decreases from the first premolar to the second molar, and no effective buccal movement is achieved for the first and second molar. Increasing the amount of molar expansion gradually increases the buccal displacement of the first molar, but decreases the buccal displacement of the premolars, and even the second premolar shows a palatal movement trend opposite to the target. Among the groups with the same amount of expansion for the target teeth in both the overall movement and the distributed movement groups, only the first premolar movement group (B1) and the posterior teeth interval alternating movement groups (C1 and C2) show buccal movement for all the target teeth. Moreover, in the posterior teeth interval alternating movement group (C), the mean buccal displacement of the target tooth is the largest (0.026 mm), greater than that in the premolar/molar distributed movement group (B, 0.016 mm) and the overall movement group (A1, 0.015 mm), but the mean palatal displacement of the anchorage posterior teeth is also the largest (Figure 3B, D).

In the anterior region, the canines all show a trend of lingual tilting movement, while the lateral incisors demonstrate a trend of labial tilting movement. The central incisors mostly show a labial tilting trend, except in the premolar movement group (B2) and the second premolar-second molar movement group (C2), where they show a palatal tilt, indicating a loss of anchorage in the anterior teeth that are not designed to move. In all overall movement groups, the displacement and difference of each anterior tooth are relatively small. However, among all groups, the posterior teeth interval alternating movement group (C) shows the least anterior anchorage loss (Figure 3C). The maximum displacement of the anterior teeth is shown in Table 4.

**TABLE 4:** Maximum displacements of anterior teeth in groups with different tooth movement patterns (Unit: mm).

Group	A1	A2	A3	B1	B2	C1	C2
Central incisor	2.06E-02	1.99E-02	2.27E-02	1.92E-02	8.61E-03	1.51E-02	6.07E-03
Lateral incisor	3.58E-02	4.04E-02	3.87E-02	3.68E-02	1.18E-02	3.05E-02	1.25E-02
Canine	2.82E-02	2.36E-02	2.47E-02	2.90E-02	8.18E-03	2.50E-02	1.15E-02

#### 3.1.2 The stress distribution of PDL

**FIGURE 4:**
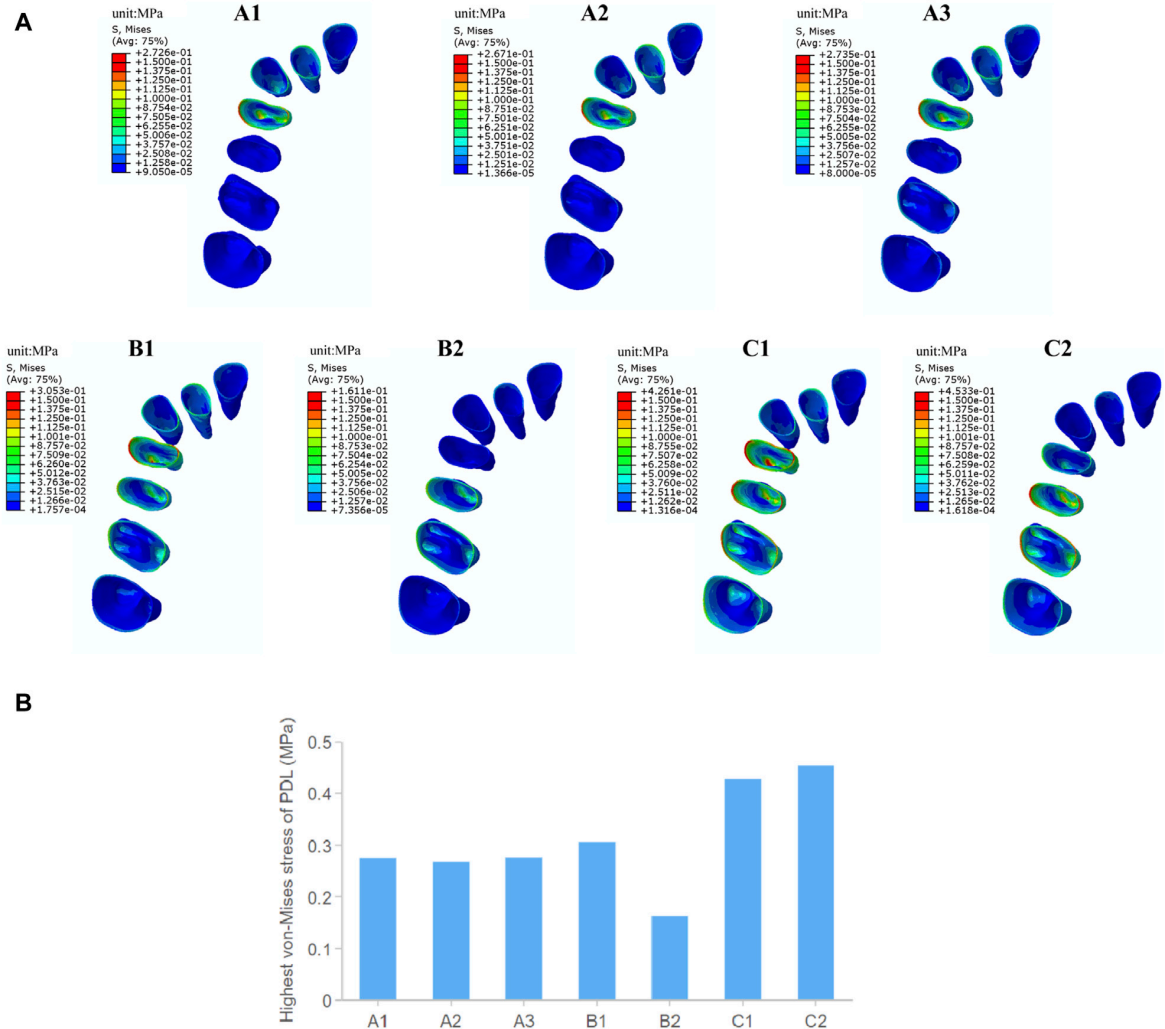
The von Mises stress of PDL in groups with different tooth movement patterns. **(A)** The von Mises stress distribution in PDL. **(B)** Highest von Mises stress value of PDL.

As shown in Figure 4, the stress distribution pattern of von Mises in the PDL is similar to the trend of tooth movement. In the overall movement groups, stress is mainly concentrated in the first premolar area, especially in the cervical and apical regions of PDL, and as the expansion amount of the molars increases, the stress on PDL of the first molar becomes more pronounced. In the distributed movement groups, stress is primarily concentrated in the cervical and apical regions of PDL of the target teeth and adjacent teeth. When the teeth to be expanded are closer to the end of the arch (in groups B2 and C2), the von Mises stress in PDL of the anterior teeth becomes less pronounced. The distributed movement groups generally show higher stress values of PDL compared to the overall movement groups, and the stress distribution is more uniform, except in cases of molar expansion. The posterior teeth interval alternating movement groups (C1C2) show the highest PDL stress values among all groups.

**FIGURE 5:**
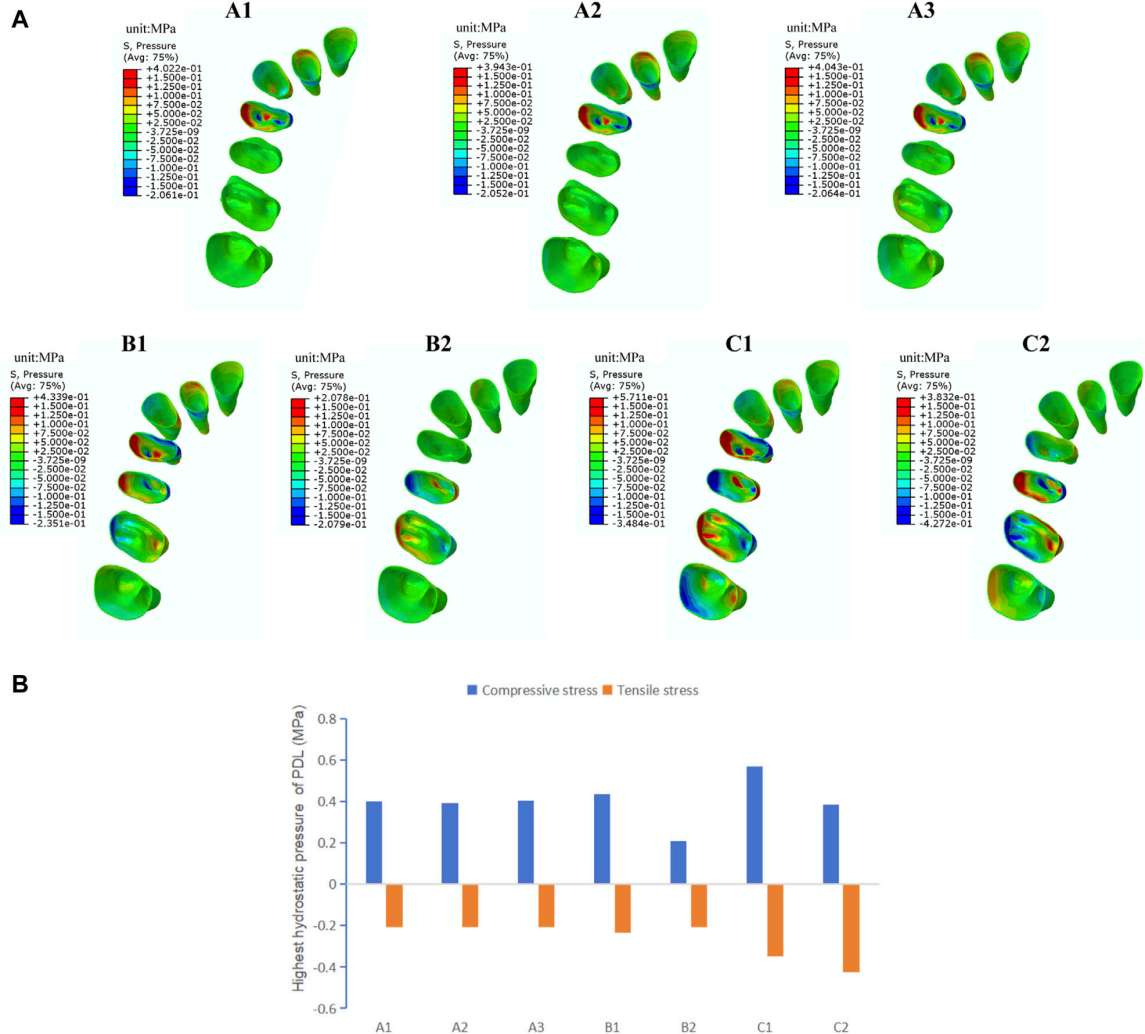
The hydrostatic pressure of PDL in groups with different tooth movement patterns. **(A)** Compressive and tensile stress distribution in PDL. **(B)** Highest compressive and tensile stress value of PDL.

Orthodontic tooth movement occurs as a biological response to mechanical stimulation of the PDL during the process of alveolar bone remodeling. According to the “pressure-tension” theory, bone apposition and resorption occur on the tensile and compressive sides of the PDL, respectively (Jia et al., 2022). As shown in Figure 5, the distribution pattern of hydrostatic pressure in the PDL during arch expansion is consistent with the trend of tooth displacement. Tensile stress concentrates mainly on the palatal side near the cervical area and the buccal root apices of the PDL of target premolars and on the palatal side near the cervical area and the palatal root apices of the PDL of target molars. Compressive stress concentrates mainly on the buccal side near the cervical area and the palatal root apices of the PDL of the target premolars, as well as on the buccal side near the cervical area and the buccal root apices of the target molars. In the distributed movement groups, especially in the posterior teeth interval alternating movement group (C), other anchorage posterior teeth also show a noticeable concentration of tensile and compressive stresses. The highest value of tensile stress occurs in the second premolar-second molar movement group (C2) with 0.43 MPa. The highest value of compressive stress occurs in the first premolar-first molar movement group (C1) with 0.57 MPa, followed by the premolar movement group (B1) with 0.43 MPa. The intergroup differences in hydrostatic pressure in the PDL are not significant in the overall movement groups. Compared to the alternating movement, the tensile stress is at a lower level, while the compressive stress is at a moderate level in the overall movement groups.

### 3.2 Effects of different aligner thicknesses

#### 3.2.1 Initial displacement and displacement trends of teeth

**FIGURE 6:**
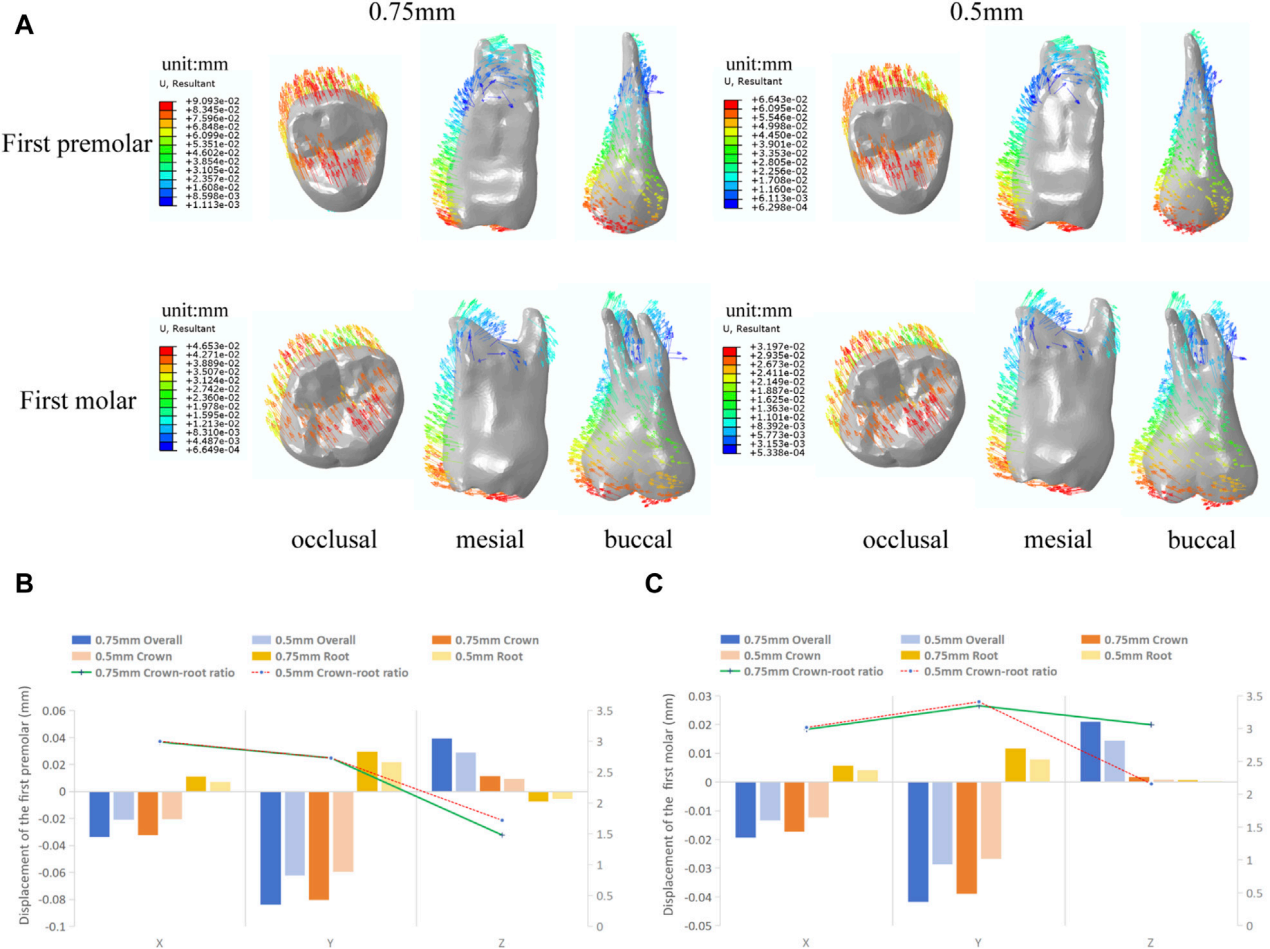
Initial displacement trends and displacement of the target teeth in groups with different aligner thicknesses. **(A)** Initial displacement trends. **(B)** Overall, crown and root displacement, and crown-root displacement ratios of the first premolar in the X, Y, and Z axis. **(C)** Overall, crown and root displacement, and crown-root displacement ratios of the first molar in the X, Y, and Z axis. (Note: Crown and root displacement are the mean of the displacement of each crown and root landmark).

As shown in Figure 6, the trend of tooth movement of the target teeth during expansion remains consistent with the loading of the aligners of different thicknesses, characterized by a tilting movement of the crowns toward the buccal side and of the roots toward the palatal side, accompanied by a tendency for distal movement of the crowns. The maximum tooth displacement produced by the 0.5 mm aligners is consistently smaller in the three axial directions compared to the 0.75 mm group. Specifically, the maximum overall tooth displacement of the first premolar and first molar in the 0.5 mm group is 73% and 69% of that in the 0.75 mm group, respectively.

#### 3.2.2 The stress distribution of PDL

**FIGURE 7:**
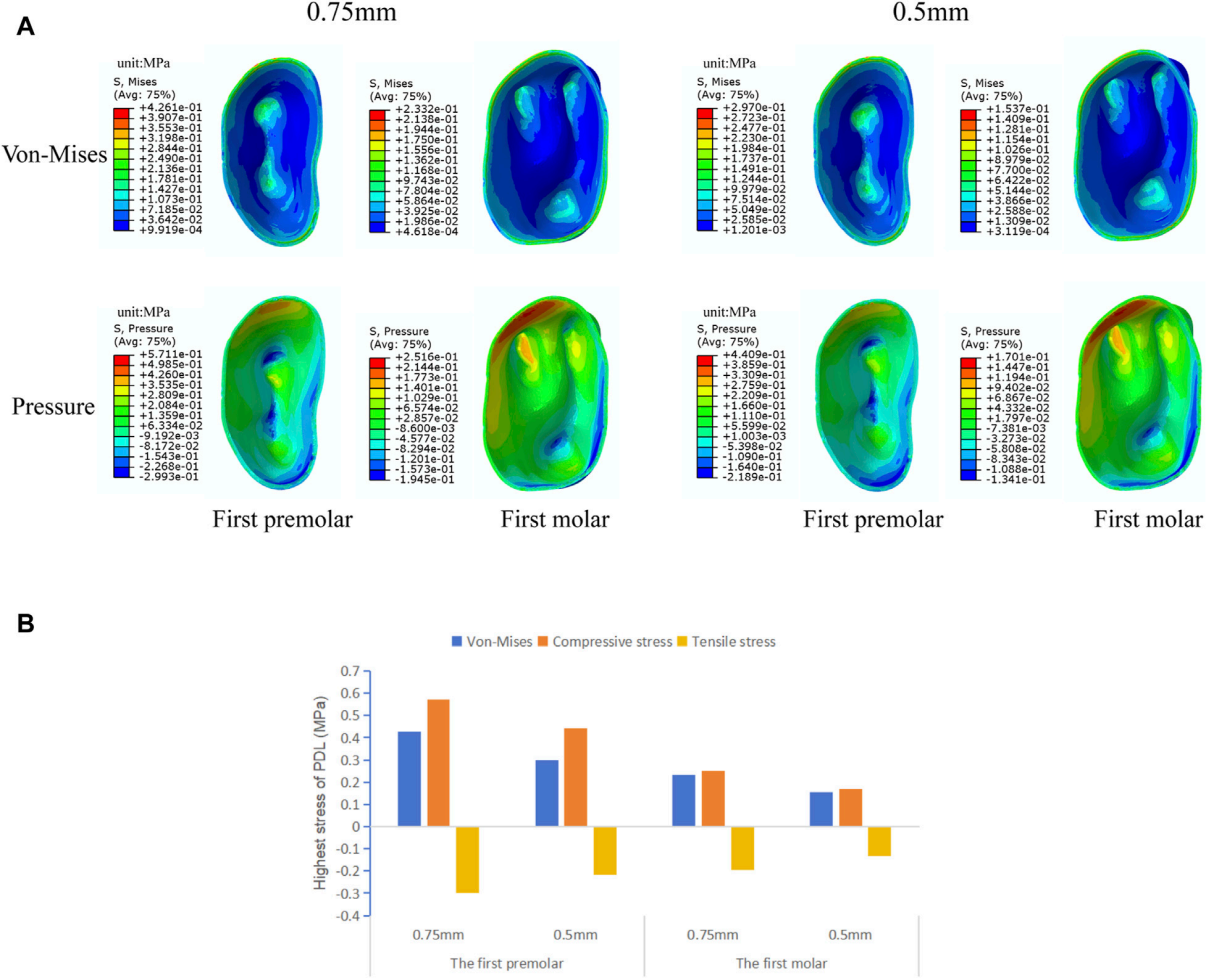
The von Mises stress and hydrostatic pressure of PDL of the target teeth in groups with different aligner thicknesses. **(A)** The von Mises, compressive and tensile stress distribution in PDL. **(B)** Highest von Mises, compressive and tensile stress value of PDL.

As shown in Figure 7, there is no significant change in the stress distribution pattern in the PDL of the target teeth under the loading of clear aligners of different thicknesses, with stress concentrations observed mainly in the cervical and apical regions of the PDL. The maximum values of von Mises stress, compressive stress, and tensile stress generated by the 0.5 mm thick aligner are all lower than those produced by the 0.75 mm aligner, with a more pronounced reduction observed particularly in the first premolar.

## 4 Discussion

Clear aligners are widely used in clinical practice due to their excellent comfort and aesthetics. They possess a completely different biomechanical system compared to fixed orthodontic appliances (Upadhyay and Arqub, 2022). By wearing clear aligners, teeth are expected to move to a series of pre-designed positions. However, achieving this goal is not always easy because plastic polymer materials cannot provide sufficient strength and elasticity to ensure that teeth move completely to the specified positions (Lombardo et al., 2017), especially during expansion, where significant tooth tipping often occurs, and the expansion efficiency in the molar region is relatively low (Bouchant et al., 2023). FEM, based on stable and reproducible models, can be used to study the biomechanical effects of orthodontic appliances and their trends. Therefore, this study compares the biomechanical mechanisms of clear aligner expansion under different tooth movement patterns and aligner thicknesses through FEM, providing valuable information for the clinical application of clear aligners in orthodontic treatment.

The results of this study showed that the buccal displacement of the teeth gradually decreased from the first premolar to the second molar when the clear aligner was used in the overall expansion of the maxillary arch width and was mainly characterized by tilting movement of teeth (Figure 3A), which is same with the majority of research findings (Bouchant et al., 2023; Zhang et al., 2023). There is no significant buccal displacement in the molar region, suggesting that the efficiency of clear aligners is lower in cases involving a large number of tooth movements, especially in the posterior region. Differences in the efficiency of arch expansion in different tooth positions may be analyzed due to a combination of factors, including root anatomy, clinical height of the crowns, cortical bone thickness, occlusal loading, and buccal soft-tissue pressure, but also in relation to the gradual decrease in the mechanical efficiency of the aligner in transmitting effective buccal forces from anterior to posterior (Haouili et al., 2020). In order to increase the efficiency of molar expansion, the amount of movement per step of the molar in the overall movement group was gradually increased. Experimental results show that the buccal displacement of the first molar gradually increases (Figure 3D), but the second molar still fails to achieve effective buccal displacement. Zhang et al. (Zhang et al., 2023) three-dimensional finite element study also shows that aligners rarely produce expansion effects on the second molars during expansion. Haouili’s Haouili et al. (2020) research suggests that the second molars are the most difficult teeth to predict and have the lowest expansion efficiency, which is related to the limited ability of aligners to apply orthodontic forces to terminal teeth. In addition, the experimental results indicate that as the molar expansion increases, the buccal displacement of the premolars decreases. This is consistent with the research results of Zhao et al. (Zhao et al., 2017), which found that the preset expansion amount of the molar significantly affects the expansion efficiency of the premolar; when the planned inter-molar width increase is ≥2 mm, the effectiveness of premolar expansion is significantly reduced.

Studies have shown that when using clear aligners to distalize molars and intrude anterior teeth, sequential molar distalization (Liu et al., 2023) and distributed anterior teeth intrusion (Liu and Hu, 2018) are more sensible than overall movement. Therefore, different distributed movement patterns were designed in this study to investigate the influence of tooth movement patterns on expansion with clear aligners. The experimental results indicate that the mean buccal displacement of target posterior teeth is greater in the premolar-molar interval alternating movement group than in the premolar/molar distributed movement group and the posterior teeth overall movement group. Thus, the movement pattern of alternating movement between premolars and molars at intervals has the highest average expansion efficiency. This finding is also supported by the experimental results of Yao et al. (Yao et al., 2023). Anchorage design in clear aligner expansion includes intra-arch anchorage, including mutual anchorage between bilateral posterior teeth and adjacent tooth anchorage. In the alternating posterior movement groups, bilateral direct adjacent tooth anchorage is achieved for the target premolars and first molars, while unilateral direct adjacent tooth anchorage is achieved for the second molars. The remaining groups have only partial adjacent tooth anchorage, resulting in less buccal displacement efficiency compared to the alternate movement groups. In addition, anterior teeth that were not designated for movement also exhibited varying degrees of displacement trends. Among them, the total displacement of anterior teeth in the posterior teeth alternate movement group was the least, but the loss of posterior adjacent tooth anchorage was the highest. Therefore, further exploration is warranted to add additional anchorage design in the clinical application of clear aligners for arch expansion.

The results of this study indicate that stress on the PDL is mainly concentrated in the cervical and apical areas. In the overall movement groups, there is a significant stress concentration in first premolar area, with compressive stress on the side where the tooth moves toward and tensile stress on the side where the tooth moves away, indicating that the stress distribution pattern in PDL is consistent with the trend of tooth movement, which is in accordance with previous research results (Jia et al., 2022). In the overall movement, the aligner first deforms in the mesial region of the first premolar, where a large stress is generated and transmitted to the teeth, with a corresponding large stress concentration in the periodontal tissues of the first premolar and adjacent canine. In the alternating movement group, stresses were widely and significantly distributed around the target and adjacent teeth (Figure 4A). Hohmann et al. (Hohmann et al., 2007) reported that if hydrostatic pressure exceeds typical human capillary blood pressure in the PDL, there is an increased risk of root resorption. Lee (Lee, 1965) has pointed out that the highest stress value that the PDL can withstand is 26 KPa. The highest stress values of PDL in experimental groups are all greater than that value. However, the simulated calculations in this study represent initial stresses, and orthodontic tooth movement is a dynamic process. The high instantaneous stresses during beginning wear of the aligners will decay exponentially to lower levels with wear without causing major damage to the periodontal tissues (Lombardo et al., 2017). Nevertheless, clinical vigilance should be maintained in areas where periodontal stress concentrations may occur to prevent periodontal tissue damage and root resorption.

Currently, the most commonly used clear aligners are 0.5 mm and 0.75 mm thick (Bichu et al., 2023). The different aligner thicknesses affect the release of orthodontic forces and inevitably influence tooth movement. Therefore, based on the results of the previous part of the experiment, we designed 0.5 mm and 0.75 mm aligner models for first premolar and first molar expansion to analyze the effect of different aligner thicknesses in clear aligner treatment. The experimental results show that during expansion with aligners of different thicknesses, there is no significant change in the trend of tooth movement, which still shows a tilting movement of the crowns toward the buccal side and of the roots toward the palatal side, accompanied by a tendency for distal movement of the crowns (Figure 6). The crown-root displacement ratios in the mesial-distal and buccolingual directions show no significant difference, indicating that increasing the aligner thickness has no significant effect on the tooth bodily movement control during expansion. Similarly, there is no significant change in the stress distribution pattern of the PDL. However, with the increase in aligner thickness, both the tooth displacement and PDL stress value increase (Figures 6, 7), which is consistent with the conclusions of Lyu (Lyu et al., 2023). This suggests that as the aligner thickness increases, the orthodontic force generated also increases, which can improve the expansion efficiency, but it also increases the load on the periodontal tissues. In orthodontic clinical practice, the efficiency of tooth movement and the condition of periodontal tissues should be comprehensively considered to select the most appropriate aligner thickness for patients.

During arch expansion with clear aligners, tooth movement occurs primarily through buccal inclination, as shown by most research (Zhou and Guo, 2020; Tien et al., 2023). The forces generated by the aligner material are limited and cannot adequately support buccal bodily movement of posterior teeth, resulting in significant tipping. Overcorrection design with torque compensation can reduce the tendency for tooth inclination. However, it is difficult for clear aligners to achieve both good torque control and efficient movement during expansion (Yao et al., 2023). Studies have shown that blind pursuit of bodily movement significantly reduces the efficiency of maxillary arch expansion (Zhang et al., 2023). Therefore, when clinically applying clear aligners, it is essential to first assess the relationship between teeth and alveolar bone, alveolar bone width, and the initial torque of the teeth. Suitable cases should be selected, and appropriate buccal root torque should be applied within the appropriate range of expansion.

The three-dimensional finite element method is one of the most effective methods for studying the biomechanics of clear orthodontic appliances, and the finite element model created in this experiment by combining the CBCT data and the intraoral optical scan data is more accurate than the tooth models created in most studies using CBCT data alone. In addition, mesh quality evaluation and convergence analysis were performed to ensure the accuracy of the study results. However, there are also some limitations in this study. As an *in vitro* theoretical simulation method, FEM mainly analyzes the initial tooth displacement trend and periodontal stress distribution, and it unavoidably simplifies the material properties, thus providing approximate solutions that do not fully reflect the remodeling of oral hard and soft tissues, occlusal loading, and other complex clinical situations. Therefore, the conclusions of finite element studies should be interpreted cautiously in the context of the clinician’s own clinical experience. Subsequent animal experiments and prospective clinical trials may be conducted to verify the validity of the method.

## 5 Conclusion

This study used the finite element method to analyze and discuss the biomechanical effects of different tooth movement patterns and aligner thicknesses on teeth and periodontal tissues during maxillary arch expansion with clear aligners. Given the limitations of this study, the following conclusions were drawn:

1) When expanding the maxillary arch with clear aligners, the main trend observed is the buccal inclination of the teeth. The efficiency of expansion gradually decreases from the first premolar to the second molar.
2) Increasing the amount of expansion per step in the molars can improve the expansion efficiency of the first molar but may decrease the expansion efficiency of the premolars.
3) The tooth movement pattern of alternating movement of posterior teeth at intervals has a higher average expansion efficiency and less anterior tooth anchorage loss compared to the distributed movement of premolars/molars and the overall movement pattern. However, attention should be paid to strengthening the design to protect the posterior tooth anchorage.
4) When expanding the maxillary arch with clear aligners of different thicknesses, there is no significant change in the trend of tooth displacement. Increasing the thickness of the aligner can increase the efficiency of expansion, but also increase the burden on the periodontal tissues.

## Data Availability

The original contributions presented in the study are included in the article/Supplementary material, further inquiries can be directed to the corresponding author.
